# Regulation of soil ammonia-oxidizing microbial community assembly by alfalfa (*Medicago sativa*) planting duration in the Loess Plateau

**DOI:** 10.3389/fmicb.2025.1517296

**Published:** 2025-06-19

**Authors:** Liangliang Li, Zhuzhu Luo, Lili Nian, Lingling Li, Yining Niu, Yaoquan Zhang, Renyuan He, Jiahe Liu

**Affiliations:** ^1^Grassland Science College, Gansu Agricultural University, Lanzhou, China; ^2^College of Resources and Environmental Sciences, Gansu Agricultural University, Lanzhou, China; ^3^Institute of Soil, Fertilizer and Water-saving Agriculture, Gansu Academy of Agricultural Sciences, Lanzhou, China; ^4^State Key Laboratory of Aridland Crop Science, Gansu Agricultural University, Lanzhou, China; ^5^College of Forestry, Gansu Agricultural University, Lanzhou, China

**Keywords:** alfalfa, planting durations, Loess Plateau, community assembly, community structure

## Abstract

To investigate the effects of long-term alfalfa planting on the structure and diversity of soil ammonia-oxidizing microbial communities, this study conducted a field experiment in the semi-arid region of the Loess Plateau. Alfalfa fields planted in 2019 (L2019), 2012 (L2012), and 2003 (L2003) were studied, with farmland corn serving as the control (CK). High-throughput sequencing was used to examine the ammonia-oxidizing microbial communities and their interactions in alfalfa with varying planting durations. The results demonstrated that alfalfa planting significantly increased the levels of total nitrogen, and organic carbon compared to CK. The gene abundance of ammonia-oxidizing archaea (AOA) and ammonia-oxidizing bacteria (AOB) increased with longer alfalfa planting durations. The ecological network analysis showed that at low planting years, species in the AOA community were mainly in a collaborative relationship, while species in the AOB community were mainly in a competitive relationship. This relationship changed at high planting years. Structural equation modeling indicated that planting duration was significantly correlated with Soil water content, total nitrogen, and ammonium nitrogen. Additionally, AOB communities were significantly positively correlated with ${NH}_{4}^{+}$-N and negatively correlated with nitrate nitrogen. Ecological null model analysis revealed that the assembly of AOA and AOB communities was primarily governed by stochastic processes, with uncertainty being a key factor in the random assembly process. Furthermore, the β-nearest taxon index (βNTI) of AOB was significantly correlated with Soil water content. This suggests that long-term alfalfa planting forms a stable soil environment, enhancing stochastic processes, which is conducive to maintaining the sustainability and stability of the artificial grassland ecosystem function.

## 1 Introduction

Alfalfa is a widely cultivated perennial leguminous herb known for its deep root system, robust growth, drought and cold tolerance, and wide adaptability. It is extensively used worldwide as a forage crop, green manure, and soil amendment (Pan et al., 2023). In China, alfalfa is not only a key livestock forage crop but also a valuable resource for ecological restoration and sustainable land use (González del Portillo et al., 2022). Alfalfa cultivation enhances soil nitrogen content through biological nitrogen fixation (Song et al., 2021), with its rhizobium-based nitrogen fixation system being one of the most efficient in nature, making it a key species for soil improvement and providing high-quality forage (Duan et al., 2019; Zhao et al., 2020). However, research indicates that alfalfa’s nitrogen fixation capacity is relatively weak during its early growth stages, and the fixed nitrogen is insufficient to support high yields (Xu et al., 2021). Therefore, there is still a significant lack of understanding regarding the nitrogen cycling microbial driving mechanisms in alfalfa planting systems, particularly the mechanisms by which planting years influence the construction of ammonia-oxidizing microbial communities.

Soil ammonia-oxidizing microorganisms are those that catalyze the oxidation of ammonia (NH_3_) to nitrite (${NO}_{2}^{-}$) and subsequently to nitrate (${NO}_{3}^{-}$) in the soil (Wei et al., 2021). These microorganisms primarily consist of ammonia-oxidizing archaea and ammonia-oxidizing bacteria, which are widely distributed in nature and play a crucial role in the ecological environment and agricultural production (Yin et al., 2018). Ammonia-oxidizing archaea and ammonia-oxidizing bacteria differ in their ecological niche, but both play critical roles in nitrification (Hu et al., 2014). Nitrification is a key process in the global nitrogen cycle, influencing not only terrestrial nitrogen cycling but also the release and leaching of nitrous oxide (N_2_O) and nitrate (${NO}_{3}^{-}$) from soils (Aryal et al., 2022). In recent years, advances in molecular biology have enabled more in-depth research on ammonia-oxidizing archaea and ammonia-oxidizing bacteria, and there has been growing scholarly attention to the effects of environmental conditions on their abundance and community structure (Ouyang and Stark, 2017; Szukics et al., 2018). Studies have shown that extended planting durations lead to changes in soil environmental conditions, such as the accumulation of soil organic matter, alterations in soil pH, and fluctuations in soil nutrient dynamics, all of which influence the survival and activity of ammonia-oxidizing microorganisms (Li et al., 2022). Additionally, prolonged planting durations result in shifts in soil microbial community structure, including variations in the types, abundance, and activity of ammonia-oxidizing microorganisms (Wang et al., 2018). Higher diversity of ammonia-oxidizing microorganisms has been linked to faster rates of soil ammonia oxidation, which promotes nitrogen uptake by plants (Kena et al., 2020). However, the diversity of these microorganisms is also influenced by soil environmental factors, such as moisture, temperature, and oxygen availability (Liu et al., 2017). Understanding the ecological characteristics of ammonia-oxidizing microbial communities in systems with varying alfalfa planting durations is crucial for enriching nitrogen cycling and optimizing artificial grassland management.

Recent studies have investigated changes in the structure and diversity of soil bacterial and fungal communities across different alfalfa planting durations, particularly in hilly and mountainous regions (Zheng et al., 2021). Most research on the rhizobium symbiosis system of alfalfa has emphasized its nitrogen fixation efficiency and the factors influencing it (Abdi et al., 2021; Duan et al., 2022). However, the role of soil ammonia-oxidizing microorganisms, which are crucial for soil nitrogen transformation and alfalfa’s nitrogen uptake, has received limited attention (Cai et al., 2021). Based on the above understanding, this study will focus on addressing the following three progressive questions: (1) How does the diversity and community structure of ammonia-oxidizing microorganisms respond to different planting years? (2) Do the relative contributions of deterministic and stochastic processes to community construction change with planting years? (3) Is there a key year threshold that leads to significant changes in the functions of ammonia-oxidizing microorganisms? By answering these questions, this study will provide important theoretical support and practical guidance for optimizing alfalfa grassland management.

## 2 Materials and methods

### 2.1 Site description

The experiment was conducted at the Gansu Agricultural University Dryland Agriculture Comprehensive Experiment Station, located in Mazichuan Village, Lijiabao Town, Dingxi City, Gansu Province (35°28′N, 104°44′E), in the central part of the Loess Plateau, a typical rain-fed agricultural region. The area receives an average annual solar radiation of 141.6 kcal/cm^2^, with 2,476.6 h of sunshine per year. The average annual temperature is 6.4°C, with an accumulated temperature ≥ 0°C of 2,933.5°C and ≥ 10°C of 2,239.1°C. The frost-free period lasts approximately 140 days. The region experiences an average annual precipitation of 400 mm and an annual evaporation rate of 1,531 mm. The soil type is loess, which is well-suited for crop growth.

### 2.2 Experimental design and soil sampling

The experiment was conducted on alfalfa grasslands, established in 2003 (L2003), 2012 (L2012), and 2019 (L2019), with adjacent farmland used as the control. Corn, the primary local crop, was planted in the control farmland. For each treatment, three replicate plots, each measuring 21 m^2^ (3 m × 7 m), were arranged in a randomized block design. The alfalfa seeding rate was 18 kg⋅hm^–2^, with a row spacing of 20 cm. Upon establishment, both nitrogen and P_2_O_5_ were applied at a rate of 105 kg⋅hm^–2^. The corn variety “Xianyu 335” was sown at a density of 52,500 plants⋅hm^–2^, with 200 kg⋅hm^–2^ of nitrogen and 105 kg⋅hm^–2^ of P_2_O_5_ applied before sowing each year. No additional fertilizers were applied during the growing season. The plots were adjacent, the terrain was flat, and edge effects were minimized. No irrigation or fertilization occurred during the growth period, and the management practices for each treatment were consistent, with mowing conducted twice annually.

In June 2020, soil samples were collected at a depth of 0–30 cm around the root zone using the five-point sampling method during the flowering period of alfalfa and the jointing stage of corn. The samples were cleared of gravel, plant residues, and other debris, thoroughly mixed, and placed in self-sealing bags. They were stored on dry ice and promptly transported to the laboratory. The soil samples were then divided into two portions: one was stored at −*80*°C for the analysis of ammonia-oxidizing microorganisms, while the other was air-dried and kept for the determination of physicochemical properties.

### 2.3 Analysis of soil physicochemical properties

Soil water content was measured using the drying method (Sanmanee and Suwannaoin, 2009), while pH was determined using the potentiometric method (Sanmanee and Suwannaoin, 2009). Total nitrogen was assessed via H_2_SO_4_ digestion followed by the Kjeldahl method (Sanmanee and Suwannaoin, 2009), and total phosphorus and available phosphorus were measured using the colorimetric method (Sanmanee and Suwannaoin, 2009). Soil organic carbon was determined using the externally heated potassium dichromate oxidation method (Sanmanee and Suwannaoin, 2009). Nitrate nitrogen and ammonium nitrogen were extracted using 2 mol⋅L^–1^ KCl and analyzed with a semi-automatic chemical discontinuous analyzer (Smart Chem AST-6500S) (Markus et al., 1985).

### 2.4 Soil ammonia-oxidizing microbial community sequencing

The soil samples for determining ammonia-oxidizing microorganisms were sent to Shanghai Meiji Biopharmaceutical Technology Co., Ltd. for analysis. Two pairs of primers, Arch-amoAF/Arch-amoAR (Francis et al., 2005) and AmoA-1F/AmoA-2R (Rotthauwe et al., 1997), were used, as shown in Table 1. Trans Gen AP221-02: TransStart Fastpfu DNA Polymerase was used for PCR, with the PCR performed on an ABI 7500 fluorescent quantitative PCR instrument. The PCR reaction system (20 μL) consisted of 10 μL of 2 × PCR mix, 0.8 μL each of upstream and downstream primers (5 μmol/L), 1 μL of template DNA, and ddH_2_O added to reach a final volume of 20 μL. The PCR reaction conditions were as follows: pre-denaturation at 95°C for 5 min, denaturation at 95°C for 45 s, annealing at 58°C (for AOA) or 60°C (for AOB) for 30 s, and extension at 72°C for 1 min over 35 cycles. After these steps, the 96-well plate containing the samples was placed in the ABI 7500 fluorescent quantitative PCR instrument for analysis. Three parallel experiments were conducted, with sterile water serving as a blank negative control.

**TABLE 1:** Primer names and primer sequences of target genes.

Gene	Primer name	Primer sequences 5′–3′
AOA	Arch-amoAF	STAATGGTCTGGCTTAGACG
	Arch-amoAR	GCGGCCATCCATCTGTATGT
AOB	AmoA-1F	GGGGTTTCTACTGGTGGT
	AmoA-2R	CCCCTCKGSAAAGCCTTCTTC

High-throughput sequencing was performed on the Illumina MiSeq PE300 × 2 platform. Paired-end reads were merged based on overlap using FLASH 1.2.1. Quality control and chimera checking of raw sequences from different samples were conducted using QIIME 1.9.1, with sequences shorter than 50 bp and erroneous reads removed. UCHIME was used for denoising and chimera filtering. Clustering of representative sequences into operational taxonomic units (OTUs) at a 97% similarity threshold was carried out using the Uparse algorithm in USEARCH11. Taxonomic annotation of AOA and AOB amoA gene OTU representative sequences (at 97% similarity) was performed with the RDP Classifier 2.1 using the Functional Genomics Resources (FGR) database and a Bayesian approach. To normalize sequencing depth, subsampling was performed using Mothur 1.30.2 based on the minimum sequence number across all samples.

Alpha diversity of the soil protozoan communities under different phosphorus addition treatments was calculated using the “vegan” and “picante” packages in R. Diversity indices included Sobs, ACE, Shannon, and Simpson, all computed at the OTU level. Beta diversity was assessed using Bray-Curtis dissimilarity, calculated with the “vegan” package in R, to evaluate differences in soil protozoan community composition under varying phosphorus addition conditions.

### 2.5 Data analysis

Operational taxonomic units (OTUs) with a Spearman correlation coefficient *r* > 0.6 and significance *P* < 0.05 among soil ammonia-oxidizing microorganisms were selected for the construction of a microbial community correlation network, using the “igraph” and “psych” packages in R software. Network visualization was performed using Gephi 0.9.2 software. The nearest taxon index (βNTI) was calculated using the “mntd” and “ses.mntd” functions in the “picante” package in R software to represent the assembly process of ammonia-oxidizing microbial communities. A βNTI value of βNTI < −2 or > 2 indicates deterministic processes, with homogeneous selection (βNTI < −2) and heterogeneous selection (βNTI > 2) dominating. Values between −2 < βNTI < 2 indicate that stochastic processes are dominant (Stegen et al., 2013). The Raup-Crick matrix based on the Bray-Curtis dissimilarity (RCbray) was calculated using the “vegan” package in R. RCbray values greater than 0.95 suggest dispersal limitation, values less than −0.95 suggest homogeneous diffusion, and |RCbray| < 0.95 indicates an ambiguous process. The structural equation model (SEM) was constructed using the R package “piecewise SEM” to further identify the key driving factors of ammonia-oxidizing microorganisms. The model fit was evaluated using multiple indicators (*P* > 0.05, AIC, Fisher’s C), and the path coefficients of the model were tested for significance with *P* < 0.05.

The I-Sanger cloud platform from Shanghai Meiji Company was used to analyze the diversity and structural composition of AOA and AOB communities. SPSS 25.0 was used to conduct one-way analysis of variance (ANOVA) and multiple comparisons (Duncan method, *P* = 0.05) to evaluate differences in physicochemical properties and microbial community diversity among treatments. The molecular ecological network was calculated using the R language and visualized with Gephi software. Redundancy analysis (RDA) between AOA and AOB at the phylum level and physicochemical factors was performed using Canoco5. The community assembly process was also calculated using R. Figures were drawn with Origin 2021, and charts were refined using Adobe Illustrator 2020.

## 3 Results

### 3.1 Soil physicochemical properties

**FIGURE 1:**
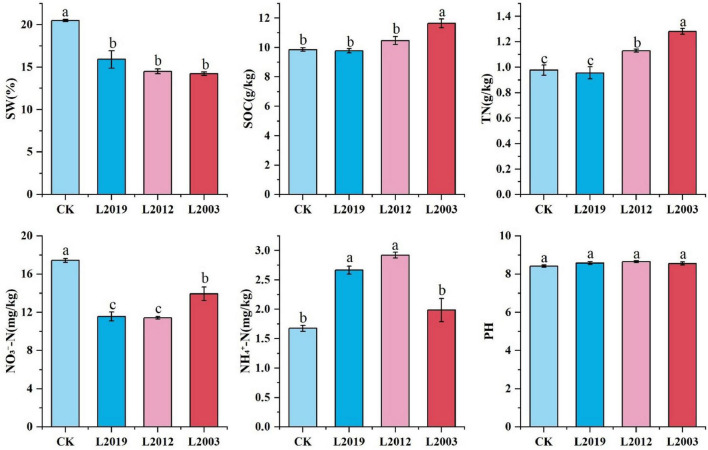
Analysis of physicochemical properties. Different lowercase letters indicate significant differences with a value of *P* < 0.05 based on the ANOVA. SOC, Soil organic carbon; TN, total nitrogen; SW, soil water content; ${NH}_{4}^{+}$-N, soil ammonium nitrogen; ${NO}_{3}^{-}$-N, soil nitrate nitrogen.

As shown in Figure 1, significant differences in soil physical and chemical properties were observed across different planting years. The soil water content in the CK treatment was significantly higher than in the alfalfa treatments (*P* < 0.05) and decreased with longer alfalfa planting durations. The total nitrogen content in the soil of the L2003 treatment was significantly higher than in other treatments (*P* < 0.05) and was 33.33% higher than in the L2019 treatment. The ammonium nitrogen content in the soil of L2019 and L2012 treatments was significantly higher than in CK (*P* < 0.05). The ammonium nitrogen content in the L2012 treatment was 46.73% higher than in the L2003 treatment. In contrast, the nitrate nitrogen content in the soil of all alfalfa treatments was significantly lower than in CK (*P* < 0.05), while the nitrate nitrogen content in the L2003 treatment was 22.07% higher than in the L2012 treatment. The soil organic carbon content in the L2003 treatment was significantly higher than in other treatments (*P* < 0.05). No significant differences in soil pH were observed among the treatments.

### 3.2 Composition of soil ammonia-oxidizing microbial communities

**FIGURE 2:**
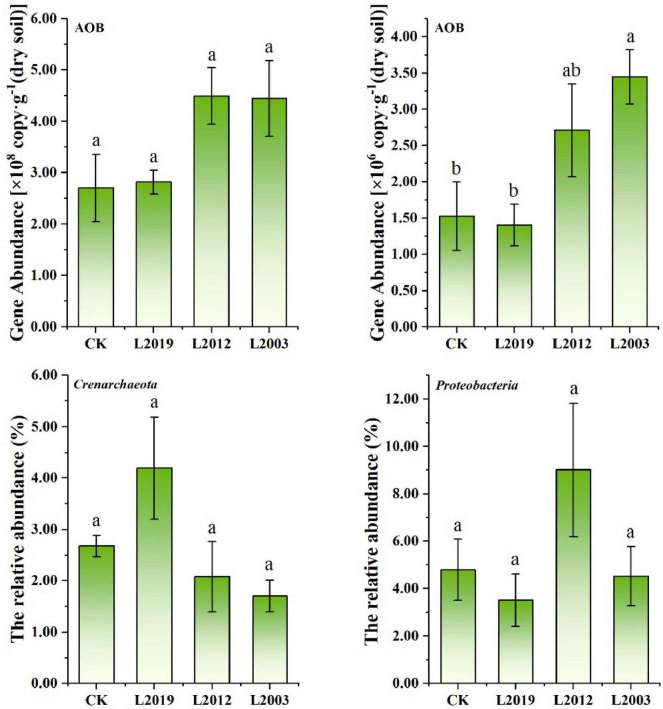
Composition of soil ammonia-oxidizing microbial community. Different lowercase letters indicate significant differences with a value of *P* < 0.05 based on the ANOVA.

The AOA and AOB gene abundance in alfalfa soils across different planting years ranged from 2.70 × 10^8^ to 4.49 × 10^8^ copies⋅g^–1^ dry soil for AOA and from 1.40 × 10^6^ to 3.45 × 10^6^ copies⋅g^–1^ dry soil for AOB. The abundance of AOA was higher than that of AOB in all treatments (Figure 2). Specifically, the AOA gene abundance in the L2012 and L2003 treatments was significantly higher than in the CK and L2019 treatment. In contrast, the AOB gene abundance in the L2003 treatment was significantly higher than in other treatments, with the abundance consistently increasing with the duration of alfalfa planting.

High-throughput sequencing analysis of ammonia-oxidizing microorganisms revealed that, at the phylum level, *Crenarchaeota* and *Proteobacteria* were dominant phyla with relative abundance greater than 1.0%. The relative abundance of *Crenarchaeota* in the AOA community was 2.66% and showed a decreasing trend with increasing planting years. In contrast, the relative abundance of *Proteobacteria* in the AOB community was 5.46%, displaying a trend of initially increasing and then decreasing as planting years increased (Figure 2).

### 3.3 Diversity of soil ammonia-oxidizing microorganisms

**FIGURE 3:**
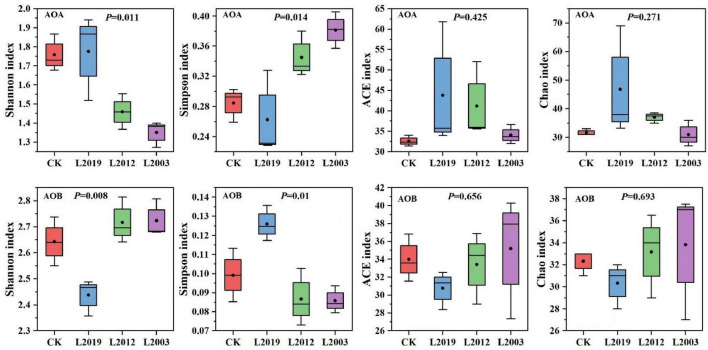
Alpha diversity index of soil ammonia-oxidizing microbial community. *P* < 0.05 indicated that there were significant differences among the treatments.

**FIGURE 4:**
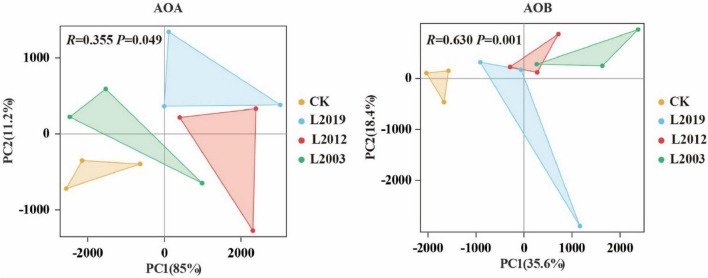
Principal component analysis (PCA) of soil ammonia-oxidizing microorganisms. *P* < 0.05 indicated that there were significant differences among the treatments.

The α diversity of the soil ammonia-oxidizing microbial community is presented in Figure 3. In the α diversity analysis of the AOA community, the Shannon, Ace, and Chao indices showed that the L2019 treatment had higher diversity than the other treatments, with a decreasing trend as planting years increased. Conversely, the Simpson index revealed that the L2019 treatment was lower than the other treatments, with an increasing trend as planting years increased. The L2003 treatment exhibited the opposite pattern. For the α diversity analysis of the AOB community, the Shannon, Ace, and Chao indices indicated that the L2003 treatment had the highest diversity, showing an increasing trend with extended planting years. The Simpson index, however, showed that the L2003 treatment was lower than the other treatments, with a decreasing trend as planting years increased. The L2019 treatment exhibited the opposite pattern. Additionally, significant differences were observed in the Shannon and Simpson indices among treatments (*P* < 0.05), while no significant differences were found in the Ace and Chao indices. Principal component analysis (PCA) was used to explore the differences in the composition of AOA and AOB communities in soils from different planting years. The PCA results (Figure 4) demonstrated that *P* < 0.05, indicating that planting duration had a significant effect on the composition of AOA and AOB communities.

### 3.4 Co-occurrence network of soil ammonia-oxidizing microbial communities

**FIGURE 5:**
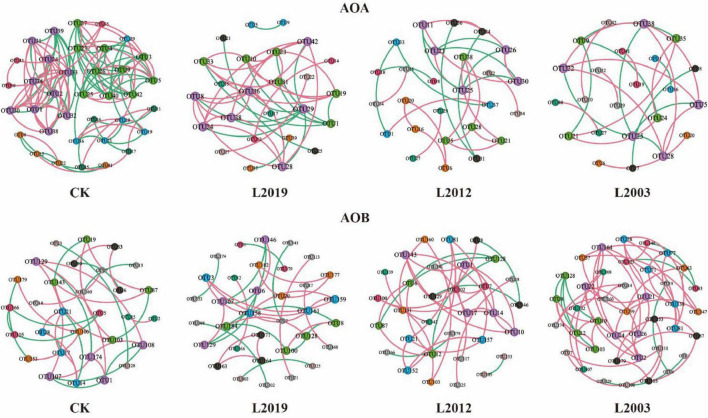
Co-occurrence network analysis of soil ammonia-oxidizing microbial communities. Node size represents the degree, indicating the number of connections to each node. Red lines signify positive connections, while green lines represent negative connections. Node colors correspond to different classifications within the microbial community, providing a visual representation of their interactions and relationships.

Different planting years exhibited significant differences in the characteristics of the soil ammonia-oxidizing microorganism co-occurrence network (Figure 5; Table 2). Analysis of the AOA co-occurrence network revealed that in the L2003 treatment, the number of edges (23), number of nodes (23), average weighted degree, and graph density were the lowest, indicating that the complexity of the AOA co-occurrence network was low in this treatment. The interactions between species were relatively simple, and the network had high modularity with clear substructures. Furthermore, the L2019 treatment showed a higher proportion of positively correlated edges and a lower proportion of negatively correlated edges compared to other treatments. These results suggest that species in the AOA community of the low planting year treatment were engaged in stronger cooperative relationships, while species in the AOA community of the high planting year treatment exhibited more competitive interactions. Analysis of the AOB co-occurrence network characteristics (Figure 5; Table 3) revealed that in the L2019 treatment, the number of edges (32), nodes (34), average weighted degree, and graph density were the lowest, indicating that the complexity of the AOB co-occurrence network in this treatment was low. The interactions between species were relatively simple, and the network exhibited high modularity with distinct substructures. Additionally, the proportion of positively correlated edges in the L2003 treatment was higher than in other treatments, while the proportion of negatively correlated edges was lower. These findings suggest that the AOB community in the high-planting-year treatment was characterized by stronger cooperative interactions.

**TABLE 2:** Topological parameters of the soil AOA co-occurrence network.

Topological parameters	CK	L2019	L2012	L2003
Node	36	25	26	23
Edge	115	42	29	23
Positive edge	53.04%	66.67%	62.07%	60.87%
Negative edge	46.96%	33.33%	37.93%	39.16%
Average weighting	3.194	1.68	1.115	1.00
Graph density	0.091	0.07	0.045	0.045
Modularity	0.68	0.619	0.801	0.73

**TABLE 3:** Topological parameters of the soil AOB co-occurrence network.

Topological parameters	CK	L2019	L2012	L2003
Node	30	34	32	40
Edge	35	32	36	61
Positive edge	57.14%	59.38%	80.56%	86.89%
Negative edge	42.86%	40.62%	19.44%	13.11%
Average weighting	1.167	0.941	1.125	1.525
Graph density	0.04	0.029	0.036	0.039
Modularity	0.834	0.869	0.843	0.856

### 3.5 Assembly patterns of soil ammonia-oxidizing microbial communities

**FIGURE 6:**
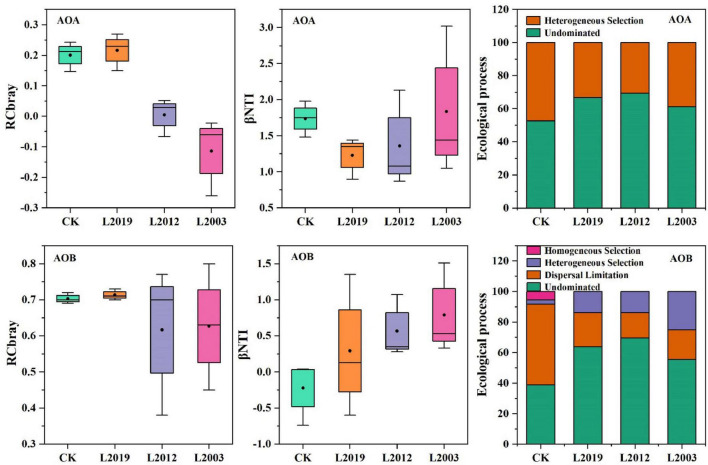
Assembly process of soil ammonia-oxidizing microbial community.

**FIGURE 7:**
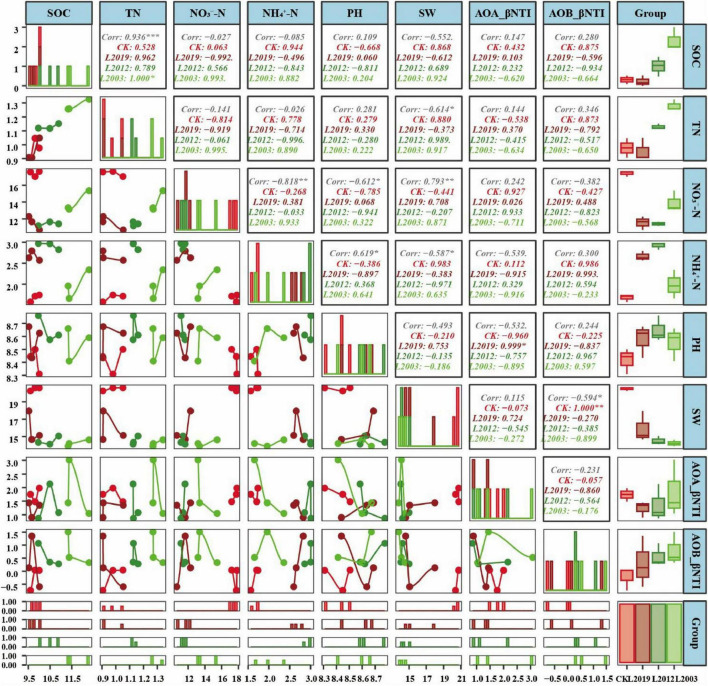
Correlation analysis between soil physicochemical factors and the nearest taxonomic unit index (βNTI) of soil ammonia-oxidizing microbial communities. The upper plot displays the correlation coefficients and *p*-values, with numbers in the boxes representing the correlation between two variables. Significance is indicated by *P < 0.05, **P < 0.01, and ***P < 0.001. Non-significant values are not marked. The middle plot shows density maps for each variable, illustrating their data distribution. Peaks along the X-axis indicate where data for each variable are concentrated. The lower plot shows line graphs for each indicator, highlighting trends over time. Additionally, histograms and box plots display the distribution of variables across different groups, similar to the density maps in the middle. A bar chart in the lower right corner represents the number of IDs in each group, with bar heights reflecting group sizes. Red corresponds to the CK treatment, dark red to the L2019 treatment, dark green to the L2012 treatment, and light green to the L2003 treatment. SOC, soil organic carbon; TN, total nitrogen; SW, soil water content; ${NH}_{4}^{+}$-N, soil ammonium nitrogen; ${NO}_{3}^{-}$-N, soil nitrate nitrogen.

To investigate the factors driving the differences in the structure of ammonia-oxidizing microbial communities in alfalfa fields across different planting years, the intrinsic assembly mechanism of the distribution patterns was analyzed using the null model. The analysis of soil |βNTI| (Figure 6) indicated that random assembly processes dominated the community assembly of both AOA and AOB in all treatments. For the AOA community, the primary random assembly process was undominated (βNTI < 2), followed by heterogeneous selection (|RCbray| < 0.95). In the AOA community assembly, the dominated was higher in the L2012 treatment compared to other treatments, while heterogeneous selection was lower. For the AOB community, the L2012 treatment exhibited a higher dominated assembly process, whereas both heterogeneous selection and dispersal limitation were lower compared to other treatments. Further correlation analysis (Figure 7) revealed that βNTI of AOB was significantly correlated with soil moisture content, while βNTI of AOA showed no significant correlation with soil physicochemical properties, indicating that the assembly of the AOA community was influenced by a wider range of soil properties.

### 3.6 Relationship between soil ammonia-oxidizing microbial communities and physicochemical factors

**FIGURE 8:**
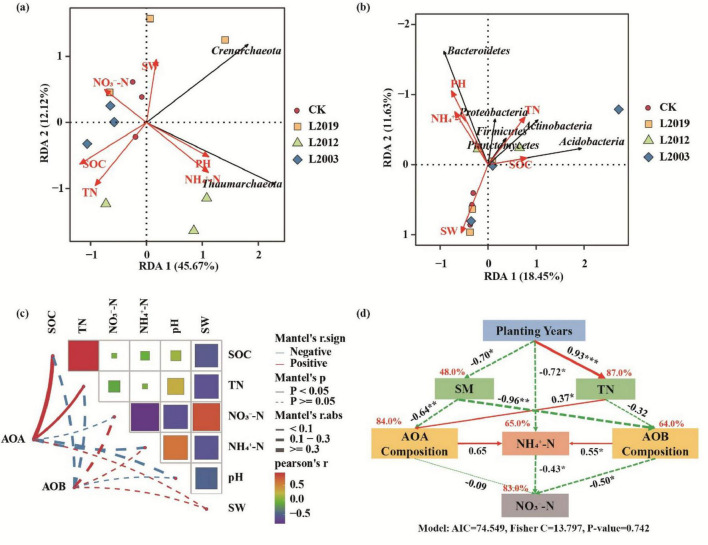
Relationship between soil physicochemical factors and ammonia-oxidizing microbial communities. **(A)** RDA analysis of the AOA community. **(B)** RDA analysis of the AOB community. **(C)** Mantel analysis of ammonia-oxidizing microorganisms and environmental factors. **(D)** Structural equation model analysis. Red solid arrows indicate significant positive correlation paths, while green dashed arrows represent negative correlation paths. The numbers on the arrows indicate standardized path coefficients. SOC, soil organic carbon; TN, total nitrogen; SW, soil water content; ${NH}_{4}^{+}$-N, soil ammonium nitrogen; ${NO}_{3}^{-}$-N, soil nitrate nitrogen.

To identify the dominant environmental factors affecting the composition of soil AOA and AOB communities, redundancy analysis was conducted using soil AOA and AOB community phylum levels as response variables and physicochemical factors as explanatory variables (Figures 8A,B). The RDA analysis of AOA communities revealed that *Thaumarchaeota* was positively correlated with pH and ${NH}_{4}^{+}$-N, while *Crenarchaeota* was positively correlated with water content. For AOB communities, RDA analysis found that *Proteobacteria*, *Bacteroidetes*, *Firmicutes*, and *Planctomycetes* were positively correlated with pH and ${NH}_{4}^{+}$-N, whereas *Actinobacteria* and *Acidobacteria* were positively correlated with total nitrogen and organic carbon. Mantel analysis (Figure 8C) showed a significant positive correlation between AOA communities and soil total nitrogen and organic carbon (*P* < 0.05), while environmental factors had no significant effect on AOB communities, suggesting that AOB communities were influenced by multiple factors. Further structural equation model analysis (Figure 8D) revealed that planting years were significantly correlated with soil water content, total nitrogen, and ${NH}_{4}^{+}$-N (*P* < 0.05). AOB communities were significantly positively correlated with ${NH}_{4}^{+}$-N (*P* < 0.05) and significantly negatively correlated with ${NO}_{3}^{-}$-N (*P* < 0.05), Additionally, ${NH}_{4}^{+}$-N was significantly negatively correlated with ${NO}_{3}^{-}$-N (*P* < 0.05), reflecting the competitive transformation process in soil nitrogen cycling, where ammonium nitrogen is converted to nitrate nitrogen through nitrification. In summary, long-term planting influences microbial community composition by altering soil properties, thereby regulating nitrogen cycling in the soil.

## 4 Discussion

### 4.1 Effects of planting years on the characteristics of ammonia-oxidizing microbial communities in alfalfa soils

This study analyzed the gene abundance and community structure of soil ammonia-oxidizing microorganisms (AOA and AOB) in alfalfa fields with different planting years, highlighting the effects of planting duration on these microbial communities. The results demonstrated that with the increase in planting years, the gene abundance of both AOA and AOB exhibited an increasing trend. This increase is likely due to the higher nitrogen and organic carbon content in alfalfa soils compared to CK, which provides sufficient nutrients for the growth and reproduction of AOA and AOB communities (Schmidt et al., 2019). Furthermore, the gene abundance of AOA was higher than that of AOB, possibly due to differences in their physiological and metabolic characteristics. AOA generally exhibits stronger adaptability and tolerance, allowing it to more efficiently utilize the energy generated during ammonia oxidation. These traits give AOA a competitive advantage, leading to its higher gene abundance (Santos et al., 2018; Schmidt et al., 2019). Additionally, the soil moisture content in alfalfa fields was lower than in CK, while total nitrogen and ammonium nitrogen contents were higher. This may be attributed to alfalfa’s nitrogen fixation ability, which increases soil nitrogen levels, and its status as a high-water-consuming crop, leading to significantly lower soil moisture in alfalfa fields compared to farmland after many years of planting (Wan et al., 2023; Zhang et al., 2023).

The relative abundance of Crenarchaeota in the AOA community was 2.66% and showed a decreasing trend with increasing planting years. This may be attributed to the relatively weak adaptability of Crenarchaeota to long-term environmental changes, leading to its decline in abundance (Devi et al., 2024). In contrast, the AOB community was composed of 8 groups, This *Proteobacteria* plays an important role in the ammonia oxidation process. The lower number of common components in the AOB community indicates relatively low diversity among ammonia-oxidizing microorganisms. This may be because *Proteobacteria*, particularly β-*Proteobacteria*, which includes key ammonia-oxidizing bacteria such as Nitrosomonas and Nitrobacter, plays a central role in oxidizing ammonia to nitrate-a crucial step in the nitrogen cycle (Su et al., 2023). Additionally, alfalfa root exudates may provide the carbon and energy required for Proteobacteria growth, further promoting their proliferation (Tang et al., 2023). The Shannon, Ace, and Chao indices for the AOA community were higher in the L2019 treatment, whereas the same indices for the AOB community were higher in the L2003 treatment. This pattern suggests that the competitive dynamics among ammonia-oxidizing microorganisms may shift with changes in planting duration. For instance, some microorganisms may have a competitive advantage in the short term, while long-term planting could favor other groups that better adapt to the evolving soil environment (Xu et al., 2023). Moreover, prolonged cultivation may exert pressure on the soil environment, leading to the suppression of certain microbial groups while promoting the growth of others (Luo et al., 2023).

Redundancy analysis of the AOA community revealed that *Thaumarchaeota* was positively correlated with pH, likely because *Thaumarchaeota* is a key microorganism in nitrification, converting ammonia to nitrate (Wright and Lehtovirta-Morley, 2023). *Thaumarchaeota* showed a positive correlation with ${NH}_{4}^{+}$-N, which is consistent with its nitrification function, as ${NH}_{4}^{+}$ serves as the direct substrate for ammonia oxidation and its abundance depends on substrate availability to sustain metabolic activity. *Crenarchaeota* exhibited a positive correlation with soil moisture, which may be attributed to the fact that increased moisture is often associated with higher soil fertility and organic matter content, factors that indirectly influence the abundance of *Crenarchaeota* (Zheng et al., 2024). Redundancy analysis of the AOB community revealed that *Proteobacteria*, *Bacteroidetes*, *Firmicutes*, and *Planctomycetes* were positively correlated with ${NH}_{4}^{+}$-N. *Proteobacteria*, a highly diverse and abundant bacterial group, includes key bacteria involved in ammonia oxidation (Yang et al., 2023). Similarly, *Bacteroidetes* and *Firmicutes*, common soil bacterial groups, play roles in organic matter decomposition and ammonia transformation (Cui et al., 2023). *Planctomycetes*, which reside in soil and aquatic ecosystems, are also involved in nitrogen cycling, including ammonia oxidation (Li Y. et al., 2023). Overall, the correlation between these microbial communities and soil environmental factors reflects their ecological functions and adaptive strategies in soil ecosystems. These relationships highlight the microorganisms’ ability to thrive in specific environmental conditions and their roles in driving essential soil biochemical processes.

### 4.2 Effects of planting years on the interaction and assembly mechanism of ammonia-oxidizing microorganisms in alfalfa soil

Molecular ecological networks not only reflect interactions between different groups within a community but also evaluate the complexity of the target community. These networks have been successfully applied to assess the impact of environmental characteristics on microbial communities (Carr et al., 2019). In this study, soil microbial co-occurrence networks differed significantly across alfalfa fields with varying planting years. The results showed strong cooperative relationships among species in the AOA community in fields with fewer planting years, while the AOB community exhibited competitive interactions. In contrast, in fields with longer planting years, the AOA community showed strong competitive relationships, whereas the AOB community displayed cooperative interactions. This shift may be attributed to increased organic matter and more complex nutrient cycling in soils with longer planting durations, which enabled species in the AOB community to adapt to nutrient-rich conditions and efficiently utilize resources through cooperation (Johnston et al., 2024). Long-term planting can also alter soil pH and redox conditions, potentially influencing the species composition of the AOA community and intensifying competition among its members (He et al., 2024). Furthermore, as planting years increase, the types and quantities of plant residues may change, impacting the structure and function of the microbial community (Sun et al., 2023). In conclusion, the duration of alfalfa planting affects the structure and function of ammonia-oxidizing microbial communities, as well as species interactions, by altering soil physical and chemical properties, nutrient cycling, plant residue composition, and the soil microenvironment. These changes result from the cumulative effects of long-term planting and soil management practices on the evolution of the soil environment.

To explore the factors driving differences in the structure of soil ammonia-oxidizing microbial communities in alfalfa fields with varying planting years, this study analyzed the intrinsic assembly mechanisms of microbial community distribution patterns based on the null model. The community assembly of ammonia-oxidizing archaea and ammonia-oxidizing bacteria in alfalfa fields treated with CK, L2019, L2012, and L2003 treatments was predominantly governed by stochastic processes. This may be attributed to alfalfa’s nitrogen-fixing properties, which reduce limiting factors for microbial activity and increase the availability of carbon and nitrogen sources to microorganisms. Consequently, stochastic processes dominate the community assembly (Li et al., 2024; Xu et al., 2023). Stochastic processes promote species coexistence, enhancing community species diversity, which is essential for ecosystem functioning. Increased diversity improves ecosystem stability and resilience, enabling the system to better withstand environmental changes and disturbances (Knelman and Nemergut, 2014). Additionally, stochastic processes buffer disturbances caused by drastic environmental shifts, supporting the stability and sustainability of ecosystem functions (Graham and Stegen, 2017; Soliveres et al., 2016). The analysis of AOA and AOB community assembly revealed differences between the L2012 and L2003 treatments, possibly reflecting the community assembly processes at different time scales. In the L2012 treatment, the relative importance of undominated processes was higher than in other treatments, suggesting that environmental conditions had a greater influence on community assembly at that time (Guo et al., 2023). Meanwhile, the relative importance of heterogeneous selection was lower, indicating reduced competitive pressure among ammonia-oxidizing microorganisms during this period (Li L. et al., 2023). In contrast, in the L2003 treatment, the relative importance of undominated processes was lower, suggesting that environmental conditions had stabilized over time or that the community had adapted, diminishing the influence of stochastic processes (Shade, 2023). The higher relative importance of heterogeneous selection and diffusion limitation in the L2003 treatment indicates the formation of more distinct niche differentiation among ammonia-oxidizing microorganisms and increased influence of dispersal limitation on community assembly (Ning et al., 2024). In summary, stochastic processes play a critical role in ecosystems by promoting species diversity and maintaining ecosystem functional stability. Under long-term alfalfa cultivation, increased stochastic processes contribute to species enrichment and the sustainability of ecosystem functions, which is vital for ecological protection and biodiversity conservation. Correlation analysis revealed a significant relationship between βNTI of AOB and soil water content, suggesting that changes in soil moisture may alter ecological niches, thereby influencing species coexistence and competition (Liu et al., 2024). Conversely, no significant correlation was found between βNTI of AOA and soil physicochemical properties, implying that the assembly of AOA communities may be influenced by multiple soil factors.

This study explored the characteristics of AOA and AOB communities and employed co-occurrence network and path analysis to reveal the effects of planting years on soil ecosystem stability in alfalfa fields. However, certain limitations remain. Although changes in community structure were identified, direct evidence regarding the ecological functions of complete ammonia oxidizers (comammox) was lacking. Future studies should integrate stable isotope probing and metagenomic approaches to specifically assess the contributions of AOA, AOB, and comammox to the nitrification process, as well as to elucidate their ecological niches in alfalfa soils.

## 5 Conclusion

The results of this study indicate that alfalfa cultivation significantly increased the soil’s total nitrogen, and organic carbon content, while also promoting the abundance of AOA and AOB as planting years progressed. The planting years significantly altered microbial interaction patterns, with AOA community species primarily exhibiting cooperative relationships and AOB community species primarily exhibiting competitive relationships during the early years of planting. However, this relationship shifted with longer planting durations. Community assembly analysis revealed that stochastic processes dominated the construction of both AOA and AOB communities, highlighting the key regulatory role of environmental factor fluctuations in controlling nitrifying microbial communities. These findings deepen our understanding of the dynamic changes in ammonia-oxidizing microorganisms in alfalfa soils.

## Data Availability

The microbial and nematode DNA sequences of the 12 soil samples were deposited in the SRA of the NCBI database under accession number PRJNA1173952.
